# Genetic Identification of Separase Regulators in *Caenorhabditis elegans*

**DOI:** 10.1534/g3.117.300298

**Published:** 2017-12-14

**Authors:** Michael Melesse, Dillon E. Sloan, Joseph T. Benthal, Quincey Caylor, Krishen Gosine, Xiaofei Bai, Joshua N. Bembenek

**Affiliations:** Department of Biochemistry, Cellular and Molecular Biology, University of Tennessee, Knoxville, Tennessee 37996

**Keywords:** separase, PPH-5, HSP-90, suppressors, *C. elegans*

## Abstract

Separase is a highly conserved protease required for chromosome segregation. Although observations that separase also regulates membrane trafficking events have been made, it is still not clear how separase achieves this function. Here, we present an extensive ENU mutagenesis suppressor screen aimed at identifying suppressors of *sep-1(e2406)*, a temperature-sensitive maternal effect embryonic lethal separase mutant that primarily attenuates membrane trafficking rather than chromosome segregation. We screened nearly a million haploid genomes and isolated 68 suppressed lines. We identified 14 independent intragenic *sep-1(e2406)* suppressed lines. These intragenic alleles map to seven SEP-1 residues within the N-terminus, compensating for the original mutation within the poorly conserved N-terminal domain. Interestingly, 47 of the suppressed lines have novel mutations throughout the entire coding region of the *pph-5* phosphatase, indicating that this is an important regulator of separase. We also found that a mutation near the MEEVD motif of HSP-90, which binds and activates PPH-5, also rescues *sep-1(e2406)* mutants. Finally, we identified six potentially novel suppressor lines that fall into five complementation groups. These new alleles provide the opportunity to more exhaustively investigate the regulation and function of separase.

Separase is a highly conserved cysteine protease required for proper chromosome segregation, and several other aspects of anaphase during both meiotic and mitotic stages of cell division (Peters *et al.* 2008). Separase proteolytic activity is inhibited during interphase and early mitosis by its pseudosubstrate inhibitor, securin (Nasmyth 2002). The protease activity of separase is critical for the cleavage of kleisin subunits of the cohesin complex (Uhlmann *et al.* 2000; Hauf *et al.* 2001). Cohesin holds sister chromatids together prior to their proper attachment to spindles and alignment on the metaphase plate preceding anaphase (Nasmyth and Haering 2009). Separase has also been implicated in various cell cycle regulatory functions. In budding yeast, separase stabilizes the anaphase spindle by cleaving the spindle and kinetochore-associated protein Slk19 (Sullivan *et al.* 2001). It is also involved in the release of the essential mitotic phosphatase Cdc14 in budding yeast (Sullivan and Uhlmann 2003). In mammalian cells, separase licenses centriole duplication (Baskerville *et al.* 2008), and a domain within its N-terminus binds and inhibits the Cyclin B-Cdk1 complex (Gorr *et al.* 2005). In mammalian cells, separase has also been shown to associate with membranes, and its depletion is associated with swelling of the *trans*-Golgi network and decreased constitutive protein secretion (Bacac *et al.* 2011). In *Arabidopsis*, separase mutant cells display mitotic failure due to defects in vesicle trafficking along microtubules, which is critical for synthesis of a cell plate during cytokinesis (Moschou *et al.* 2013). Therefore, there are numerous functions of separase during the cell cycle, and how each is regulated has not been fully elucidated.

In *Caenorhabditis elegans*, separase (SEP-1) is known to regulate multiple cell cycle events beyond its chromosome segregation functions (Bembenek *et al.* 2007, 2010). RNAi-mediated knockdown of *sep-1* leads to loss of chromosomal segregation (Siomos *et al.* 2001). Separase has been demonstrated to regulate cell cycle-related membrane transport events critical for both cytokinesis and embryonic development. During meiosis, the *C. elegans* eggshell is formed around a fertilized embryo to prevent polyspermy and provide both mechanical as well as osmotic protection for the developing embryo (Olson *et al.* 2012; Stein and Golden 2015). Formation of the eggshell is dependent on the progression of the embryonic cell cycle and requires cargo release via cortical granule exocytosis, which occurs during anaphase I (Bembenek *et al.* 2007). Importantly, separase localizes to cortical granules and is required for their exocytosis during anaphase, independently of chromosome segregation.

Various separase mutants have been identified in budding yeast, mouse, and human cells. Many of these mutants compromise the protease function of separase and directly affect its role during chromosome segregation. Interestingly, the hypomorphic *C. elegans* separase mutant *sep-1(e2406)*, originally isolated by David Livingstone in a screen for temperature-sensitive mutants defective in cell division (Siomos *et al.* 2001), is a partial separation-of-function allele. *sep-1(e2406)* is a missense mutation (C450Y) in the poorly conserved N-terminal region of separase, and has minimal effect on the chromosomal segregation role of separase but significantly diminishes cortical granule exocytosis. In embryos, SEP-1(e2406) can be observed on the spindle, similar to the wild-type protein, but shows reduced localization to cortical granules and results in a lower number of exocytic events. Another separase mutant (*sep-1(ax110)*) is a nonconditional allele that also results in minimal chromosome segregation defects and leads to cytokinesis failure (Richie *et al.* 2011). This allele is a missense mutation (H738P) in the protease domain of SEP-1 that is maternal effect embryonic lethal. These alleles potentially provide a unique opportunity to learn more about the membrane trafficking functions of separase.

Previous attempts to learn more about separase regulation used *sep-1(e2406)* to identify the PPH-5 phosphatase as a suppressor of separase (Richie *et al.* 2011). This effort screened 1.0 × 10^5^ genomes and identified three suppressors, including one *pph-5* allele, *pph-5(av101)*, an intragenic *sep-1* (L556F) mutant, and another mutant that maps to LG III. *pph-5* does not appear to be essential and mutations in *pph-5*, as well as RNAi-mediated knockdown, rescues *sep-1(e2406)* (Richie *et al.* 2011), suggesting that *pph-5* is a negative regulator of separase function. *sep-1(e2406)* suppression by *pph-5* (*RNAi)*, as well as by an in-frame deletion *pph-5* allele (*pph-5(tm2979)*), show that suppression is due to a loss, rather than an alteration, of *pph-5* function. *pph-5(av101)* is a missense mutation (P375Q) that does not suppress *sep-1(e2406)* at 24°, but does suppress at 20°, which is the minimum temperature at which embryonic lethality of *sep-1(e2406)* is fully penetrant. However, *pph-5(av101)* was effective in suppressing *sep-1(ax110)* at all tested temperatures (Richie *et al.* 2011). This observation suggests that there might be underlying differences in the effects of these SEP-1 mutations on separase function.

PPH-5 is a widely conserved phosphatase that contains N-terminal tetratricopeptide repeats (TPRs) and a C-terminal phosphatase domain. PP5, originally identified as a regulator of a variety of cellular signaling pathways including glucocorticoid receptor signaling, displays low phosphatase activity when purified due to the autoinhibitory role of its TPR domain (Chen *et al.* 1996). Interestingly, PP5 binds CDC16 and CDC27, components of the Anaphase Promoting Complex/Cyclosome (APC/C) (Ollendorff and Donoghue 1997). The APC/C is an E3 ubiquitin ligase required for the activation of separase at the metaphase-to-anaphase transition, and is regulated by phosphorylation (Kraft *et al.* 2003; Chang and Barford 2014; Musacchio 2015). The precise mechanism by which PPH-5 regulates separase is unknown, but these findings suggest that it may be an important regulator of the metaphase-to-anaphase transition.

One of the well-studied regulatory pathways of PPH-5 is its interaction with the molecular chaperone HSP-90. The crystal structure of auto-inhibited human phosphatase 5 (PP5) shows that access to the enzyme active site is blocked by a combination of the TPR domain and C-terminal *a*J-helix (Yang *et al.* 2005). HSP-90 binds the TPR domain of PPH-5 to release auto-inhibition and promote phosphatase activity toward protein substrates (Haslbeck *et al.* 2015). HSP-90 consists of three highly conserved domains and binds its client proteins via its middle domain, while it binds cochaperones via its C-terminal domain (Schopf *et al.* 2017). The very C-terminal MEEVD motif is critical for HSP90 interaction with TPR domain-containing proteins like PP5. As a major protein chaperone, HSP-90 is known to bind multiple proteins (Haslbeck *et al.* 2013). Available HSP-90 mutants, as well as RNAi in *C. elegans*, cause penetrant pleiotropic phenotypes with the null allele (*hsp-90(nr2081)*) resulting in larval lethality (Birnby *et al.* 2000; Inoue *et al.* 2006; Gillan *et al.* 2009; Gaiser *et al.* 2011). To our knowledge, there is no evidence linking HSP-90 to the regulation of separase in any system.

In this paper, we present the results of a genetic suppressor screen aimed at uncovering regulators of separase. We identified intragenic suppressors, *pph-5* mutants, a novel *hsp-90* allele, and unknown alleles that fall into five complementation groups. These suppressors may provide important insight into separase regulation and function.

## Materials and Methods

### Mutagenesis and selection

Strains were maintained as described (Brenner 1974). *sep-1(ax110)* screen: *sep-1(ax110)*/*hT2* [*bli-4(e937) let-?(q782) qIs48 (Pmyo-2*::*gfp*; *Ppes-10*:: *gfp*; *Pges-1*::*gfp)*] (I, III) worms were synchronized by bleaching with hypochlorite and grown to L4. *hT2* (I, III) acts as a balancer (Zetka and Rose 1992). Mutagenesis was performed by incubating worms with 0.5 mM ENU for 4 hr at 25° (De Stasio and Dorman 2001) and recovering in 50 ml of M9 overnight at 15°. Next, 30 P_0_s were plated on each of 81 100 mm plates, transferred to 25°, and incubated. After one generation, 50 unbalanced F_2_ progeny (nongreen pharynx as observed under a dissecting microscope and illuminated by a handheld blue laser), which should be homozygous for *sep-1(ax110)*, from each of the 81 100 mm plates were moved onto 60 mm Nematode Growth Medium (NGM) petri plates seeded with OP50, an *Escherichia coli* strain that is auxotrophic for uracil, and checked for fertility. From each nongreen F_3_-producing plate, at least six plates of nongreen animals were cloned and genotyped. Candidate suppressed lines were confirmed to be homozygous for *sep-1(ax110)* and sequenced for mutations at the *pph-5* locus.

*sep-1(e2406)* screen: homozygous *sep-1(e2406)* worms were synchronized by bleaching with hypochlorite and grown to L4. Worms were mutagenized with 0.5 mM ENU in M9 for 4 hr and recovered in 50 ml of M9 for 1 hr at 15°. Next, 100 mutagenized worms were moved to each of 60 Modified Youngren’s Only Bacto-peptone plates and incubated at 15°. P_0_s worms were moved to new plates daily. The number of F_1_ worms on each plate was estimated and plates were grown for multiple generations at 15°. These plates were then chunked and incubated at 20°, and the worms allowed to produce offspring. Plates that yielded embryos were cloned and backcrossed to *sep-1(e2406)* for multiple generations.

### Identification of suppressor mutations

#### Genotyping:

*sep-1(ax110)*; primers (oASP-UTK-3 and oASP-UTK-4, Supplemental Material, Table S2 in File S1) were used to amplify a *sep-1* fragment by PCR. The PCR product was then digested with a restriction enzyme (*Sac*II), the recognition site for which is introduced by the *sep-1(ax110)* mutation. The *sep-1(e2406)* allele was genotyped by sequencing a PCR fragment amplified using a pair of primers (oASP-UTK-34 and oASP-UTK-29) and sequenced with oASP-UTK-7.

#### PCR and sequencing:

PCR primers were used to amplify the locus of interest from worm lysates. PCR products were then gel purified and sequenced. Three PCR fragments of *sep-1*, five of *pph-5*, and two of *hsp-90* were amplified, spanning across each gene. Primers (Tables S2–S4 in File S1) used for PCR and Sanger sequencing of *sep-1*, *pph-5*, and *hsp-90* loci are listed in supplemental tables (Tables S2–S4 in File S1).

### Characterization of suppressed lines

#### Hatching assay:

Four P_0_ L4 larvae were placed in each of 35 mm OP50 NGM plates and allowed to lay embryos for 24 hr at the experimental temperature (15°, 20°, or room temperature). Worms were then transferred to new plates and returned to experimental temperature to continue laying embryos for 24 hr. The number of embryos and hatched animals on overnight plates was counted on each plate, and plates were incubated for 24 hr. The following day, the number of unhatched embryos or hatched larvae was counted, and percent hatching was quantified.

#### RNAi feeding:

Unless otherwise stated, five L1 stage worms per strain were fed at 20° (Timmons *et al.* 1998). Animals were moved to new RNAi feeding plates after reaching the L4 stage, and hatching was quantified daily for 48 hr. Worms at the L1 stage were moved onto NGM plates with ampicillin and isopropyl-β-D-thiogalactopyranoside, which were seeded with HT115(DE3) bacteria carrying RNAi feeding constructs, for 24 hr. Worms were then moved onto new RNAi feeding plates daily and hatching embryos were counted.

#### Western blot analysis:

Two-hundred young adult worms were grown at 20° on 100 mm OP50-seeded plates for one generation and collected by washing in M9 buffer. Each worm pellet was resuspended in 1× SDS loading buffer (2 μl/mg of pellet) and heated in a microwave (4 × 20 sec with 1 min cooling). Lysates were then centrifuged (15,000 × RCF, 10 min) and supernatant was transferred into new tubes. Next, 10 µl of worm lysate was loaded per well and analyzed by standard western blot. SEP-1 was detected by using a polyclonal rabbit antibody (Richie *et al.* 2011) at a dilution of 1:750. Actin was detected using the mouse monoclonal antibody C4 from Millipore (Temecula, CA) at a dilution of 1:5000. Secondary antibodies used were anti-rabbit 700 and anti-mouse 700 from Li-Cor. Quantification was done using Image Studio software. Actin was used to normalize signals between lanes. All antibody incubations were done in the presence of 5% (w/v) nonfat milk.

#### Complementation tests:

Twenty-five males generated using *him-5 RNAi* bacterial feeding for each strain were mated with five hermaphrodites on unseeded NGM plates and incubated at 20° for 24 hr. Mated worms were then moved to OP50-seeded 60 mm plates and allowed to lay F_1_ embryos at 15°. Once F_1_ worms reach L4 stage and the presence of ∼50% male animals was observed, indicative of successful mating, four L4 hermaphrodites in triplicate were moved to OP50-seeded 35 mm NGM plates and incubated at 20°. Viability of F_2_ embryos was determined.

### Data availability

All strains are available upon request. The authors state that all data necessary for confirming the conclusions presented in the article are represented fully within the article.

## Results and Discussion

### Identification of suppressors

**Figure 1:**
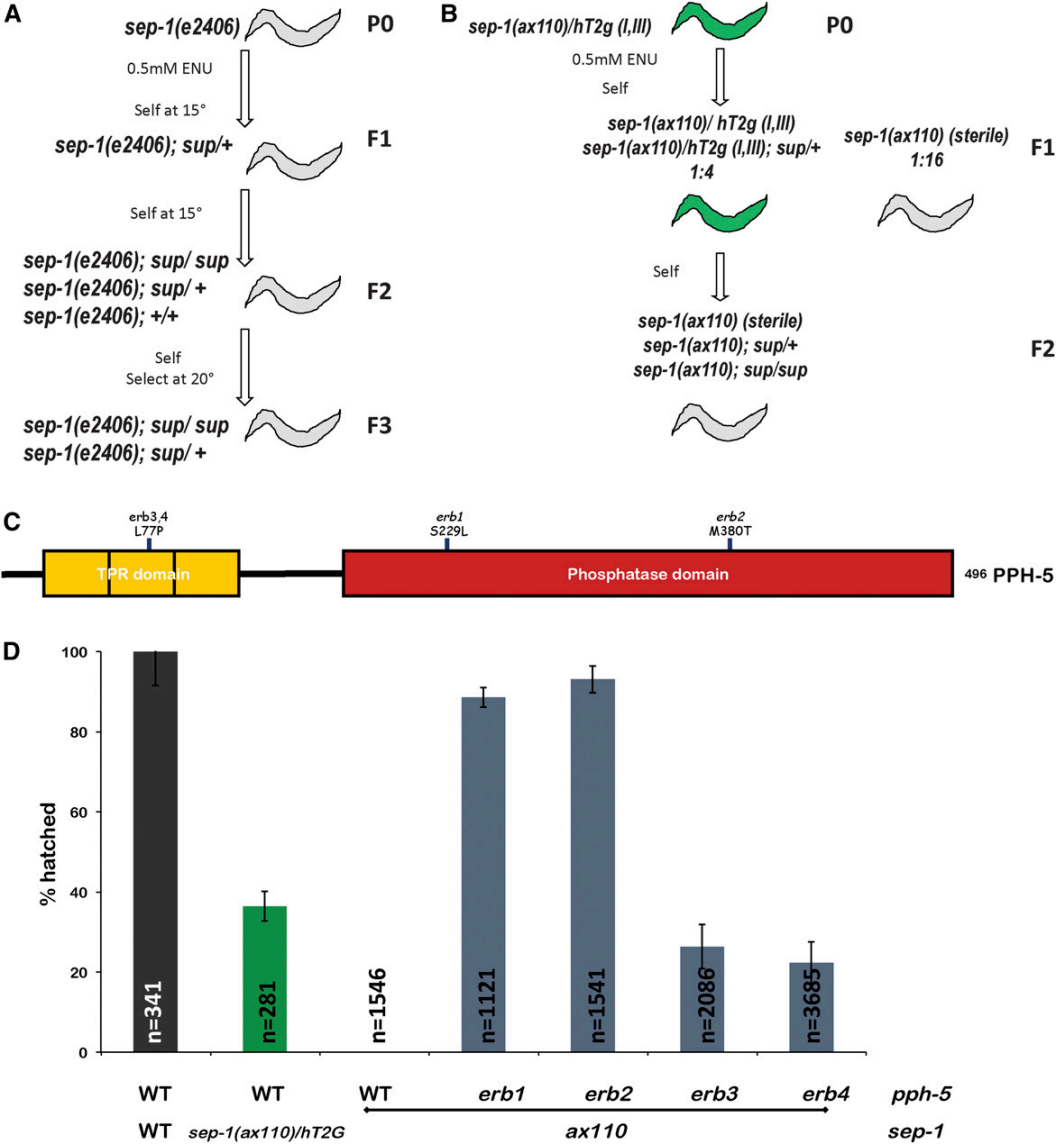
Isolation of suppressors of *sep-1(ax110)* and *sep-1(e2406)*. (A) Schematic for the isolation of lethality-suppressing mutants in the temperature-sensitive *sep-1(e2406)* background via ENU mutagenesis. (B) Schematic for the isolation of suppressor mutants in the *sep-1(ax110)* background via ENU mutagenesis. The mutant is maintained over the *hT2g* balancer and homozygous *sep-1(ax110)*, with suppressor mutations selected for by monitoring the loss of *hT2g*. The low hatching observed in the *hT2*-balanced worms is due to rescued viability (6/16) caused by aneuploidy resulting from *hT2* chromosomal segregation. (C) Protein diagram of mutations in *pph-5* that rescue the nonconditional separase mutant *sep-1(ax110)*. (D) Mutations in *pph-5* rescue nonconditional *sep-1(ax110)* mutants. *sep-1(ax110)* homozygotes carrying mutations in the phosphatase domain, *erb1* (S229L) and *erb2* (M380T), of PPH-5 have lower embryonic lethality relative to those carrying mutations in the TPR domain *erb3/4* (L77P) (*n* = number of embryos). ENU, N-ethyl-N-nitrosourea; TPR, tetratricopeptide repeat; WT, wild-type.

To identify genes that regulate separase function, we performed ENU mutagenesis screens for suppressors of two separase mutants, *sep-1(ax110)* and *sep-1(e2406)* (Figure 1A). We first screened for suppressors of the nonconditional *sep-1(ax110)* mutant (Figure 1B), which introduces a point mutation in the protease domain of SEP-1(H738P) and is maternal effect embryonic lethal. We postulated that this separase allele might be differentially impaired relative to the temperature-sensitive *sep-1(e2406)* allele, which introduces a mutation in the TPR-like domain (C450Y) and might be suppressed by a different set of mutations. A previous screen identified suppressors of *sep-1(e2406)* (Richie *et al.* 2011). Our suppressor screen identified four independent suppressors of *sep-1(ax110)*, all of which were *pph-5* mutants (*erb1(S229L)*, *erb2(M380T)*, *erb3(L77P)*, and *erb4(L77P)*) from 56,404 genomes screened (Figure 1, C and D). This is consistent with a previous finding that *sep-1(ax110)* is completely rescued by loss of *pph-5* (Richie *et al.* 2011). Our screen may suffer from inefficient isolation of nongreen progeny and not testing entire broods, which may have reduced the number of identified suppressors. Therefore, we focused our efforts toward identifying suppressors of *sep-1(e2406)*.

The *sep-1(e2406)* mutation results in a temperature-sensitive maternal effect embryonic lethality. When L4 animals are shifted to 20°, the lowest temperature at which lethality is fully penetrant, *sep-1(e2406)* hermaphrodites lay 100% dead embryos. *sep-1(e2406)* embryos are unable to perform cortical granule exocytosis and fail to build an eggshell when maintained at 25° (Bembenek *et al.* 2007; Richie *et al.* 2011). We utilized an ENU mutagenesis approach to isolate suppressors of *sep-1(e2406)* that result in viable F3 progeny at the restrictive temperature of 20° (see *Materials and Methods*) (Figure 1A). This approach yielded a total of 68 independent suppressor lines from a total of 9.6 × 10^5^ haploid genomes (as determined by counting the approximate number of mutagenized F1 progeny). Each suppressor line was cloned and backcrossed twice with the original *sep-1(e2406)* line to reduce nonsuppressing background mutations, and homozygotes were isolated. A candidate gene sequencing approach was utilized to identify suppressor mutations within *sep-1*, *pph-5*, and *hsp-90* [formerly known as *daf-21*, which is known to bind and activate PPH-5 (Haslbeck *et al.* 2015)] (see *Materials and Methods*). We also isolated six lines with novel unknown mutations belonging to at least four complementation groups.

### Intragenic suppressors of sep-1(e2406) are exclusively N-terminal

**Figure 2:**
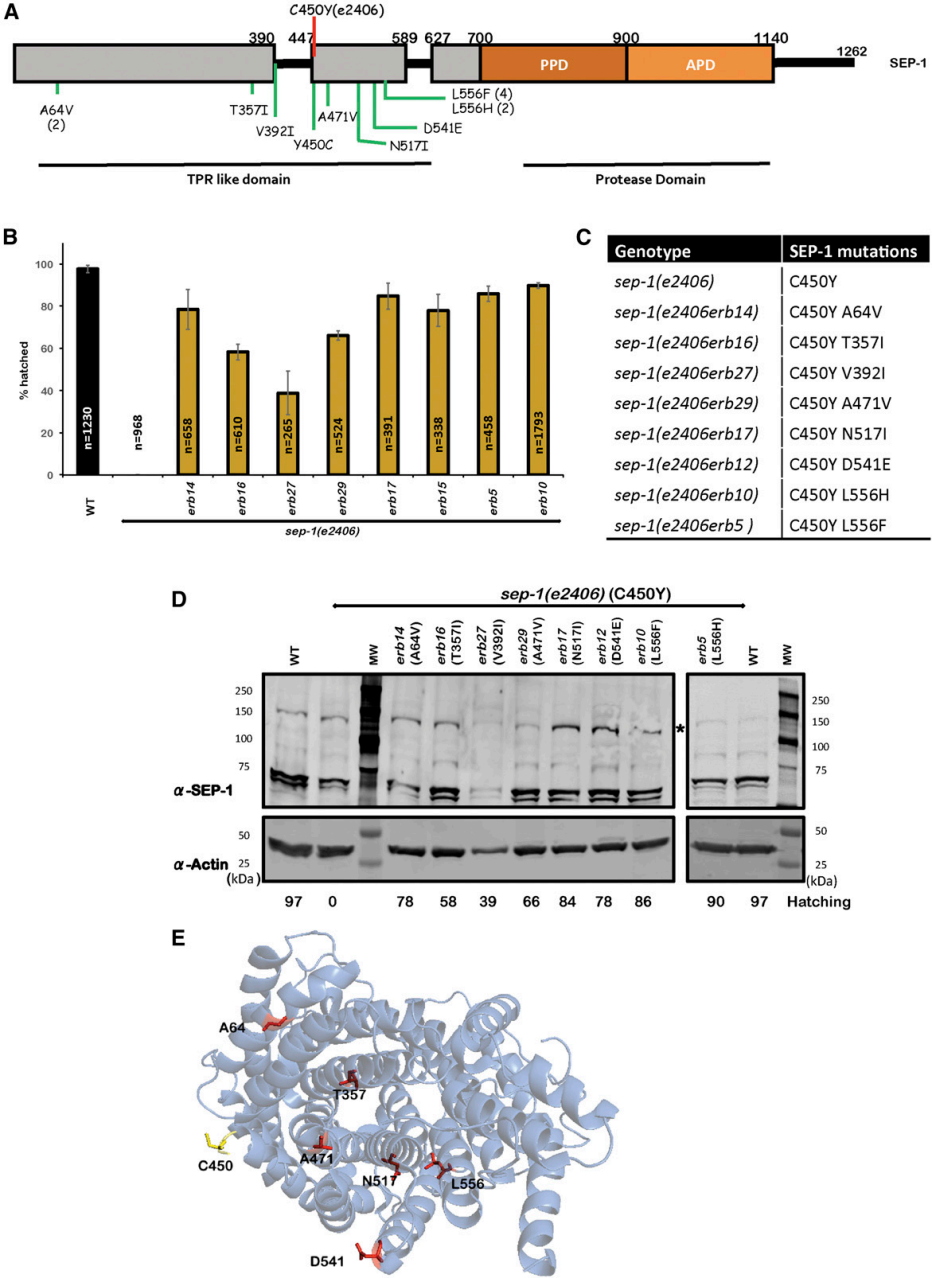
Intragenic mutations suppress *sep-1(e2406)*. (A) Protein diagram illustrating suppressing alleles of SEP-1. The causative *e2406* mutation (C450Y) is depicted with a red line. Novel suppressor mutations are in green on the protein diagram and are exclusively in the N-terminal TPR-like domain of the protein. Numbers in parentheses following a mutation indicate the number of times each suppressor was identified. (B) Embryonic lethality assays demonstrate that each suppressing mutation restores viability to *sep-1(e2406)* worms at 20° (*n* = number of embryos). (C) Table listing gene mutations and the resulting missense mutations in SEP-1. (D) SEP-1 is detectable by western blot in animals carrying *sep-1(e2406)*-suppressing mutations. Asterisk indicates SEP-1 (144 kDa). Actin was used as a loading control. Hatching values, rounded to the nearest percent, are shown below. (E) Cryo-EM structure (PDB 5MZ6) illustrating the N-terminal TPR-like domain of SEP-1. The residue mutated in *sep-1(e2406)* (C450) is shown in yellow and suppressor mutations are illustrated in red. Mapping of mutated residues onto the structure illustrates that they are distributed throughout the N-terminus. APD, active protease domain; EM, electron microscopy; PPD, pseudoprotease domain; TPR, tetratricopeptide repeat; WT, wild-type.

There were 14 independent suppressor lines identified as intragenic *sep-1(e2406)* suppressors. All intragenic *sep-1(e2406)* suppressors resulted in missense mutations within the N-terminal region of SEP-1 and none were found in the catalytic domain of the protein (Figure 2A). Some mutations were identified from multiple independent lines, suggesting that the identification of intragenic suppressor mutations was near saturation. Interestingly, mutation in lysine 556 was identified in six lines.

The types of missense mutations observed include ones that increase the size of amino acid side chains while preserving charge (A64V, A392I, A471V, and D541E). We also found mutations that remove charged side chains and introduce hydrophobic residues (T357I and N517I). The residue most frequently mutated was L556 and both changes we observed result in the introduction of aromatic side chains (L556F and L556H, Figure 2A and Figure S1 in File S1). It is also notable that L556F was previously identified as an intragenic *sep-1(e2406)* suppressor (Richie *et al.* 2011). We find that multiple residues in the N-terminus can be changed to restore function to the *sep-1(e2406)* mutant and restore viability (Figure 2, B and C).

One possible mechanism of suppression is that these mutations affect the stability of separase. To address this, we performed western blotting analysis of SEP-1 abundance in each of the suppressed lines. The separase protein is detectable in adult worms (Figure 2D), showing that proteins carrying suppressor mutations are expressed. Quantification of SEP-1 abundance, using actin as a loading control, shows that the original SEP-1(e2406) mutant protein is 70% as abundant as wild-type SEP-1. The three most effective rescuing mutations [*erb17* (N517I), *erb10* (L556H), and *erb5* (L556F)] have varying levels of expression. SEP-1(erb10) is expressed at 70% of wild-type, whereas SEP-1(erb5) and SEP-1(erb17) are 1.2-fold and twice as abundant as wild-type, respectively. SEP-1(erb12) (D541E) is expressed at twice the level of SEP-1(erb14) (A64V), but suppresses to a similar extent. Therefore, several mutants do not show a clear correlation between protein abundance and rescuing ability. However, we do observe that the least abundantly expressed mutant, SEP-1(erb27) (V392I), is the least effective suppressor. These observations suggest that these mutations do not simply affect protein levels, but may affect separase structure and function.

To gain more insight into these mutations, we mapped mutated suppressor residues onto the recently published Cryo-EM structure of SEP-1 in complex with its pseudosubstrate inhibitory chaperone IFY-1 (securin) (PDB 5MZ6, Boland *et al.* 2017). This analysis reveals that there is no clustering of mutated residues to any specific surface in the TPR-like domain of the N-terminus (Figure 2E). C450, the residue mutated in SEP-1(e2406), is at the edge of helix 16 and part of an unstructured loop containing ∼60 amino acids between helix 15 and 16 of the TPR-like domain. The effects of C450Y mutation on the structure of the TPR-like domain have not been elucidated, but there is the potential that the introduction of a large aromatic residue on this solvent-exposed loop may be unfavorable and could lead to a structural rearrangement of the SEP-1 N-terminal domain. The residues mutated in suppressed lines are found on helices not near C450, facing the interior of the protein, and are likely involved in intramolecular interactions (Figure S1 in File S1). These mutations have the potential to introduce new intramolecular interactions, leading to improved structural stability of the SEP-1 TPR-like domain, which may be disrupted in SEP-1(e2406). It is important to consider that the separase Cryo-EM structure represents a securin-bound fold of the enzyme, which is inactive. The active conformation of separase might bring these key residues into more obvious functionally relevant orientations. Our analysis indicates that we have identified multiple gain-of-function mutants that restore *sep-1(e2406)* function.

### pph-5 mutants are the most frequently identified sep-1(e2406) suppressors

**Figure 3:**
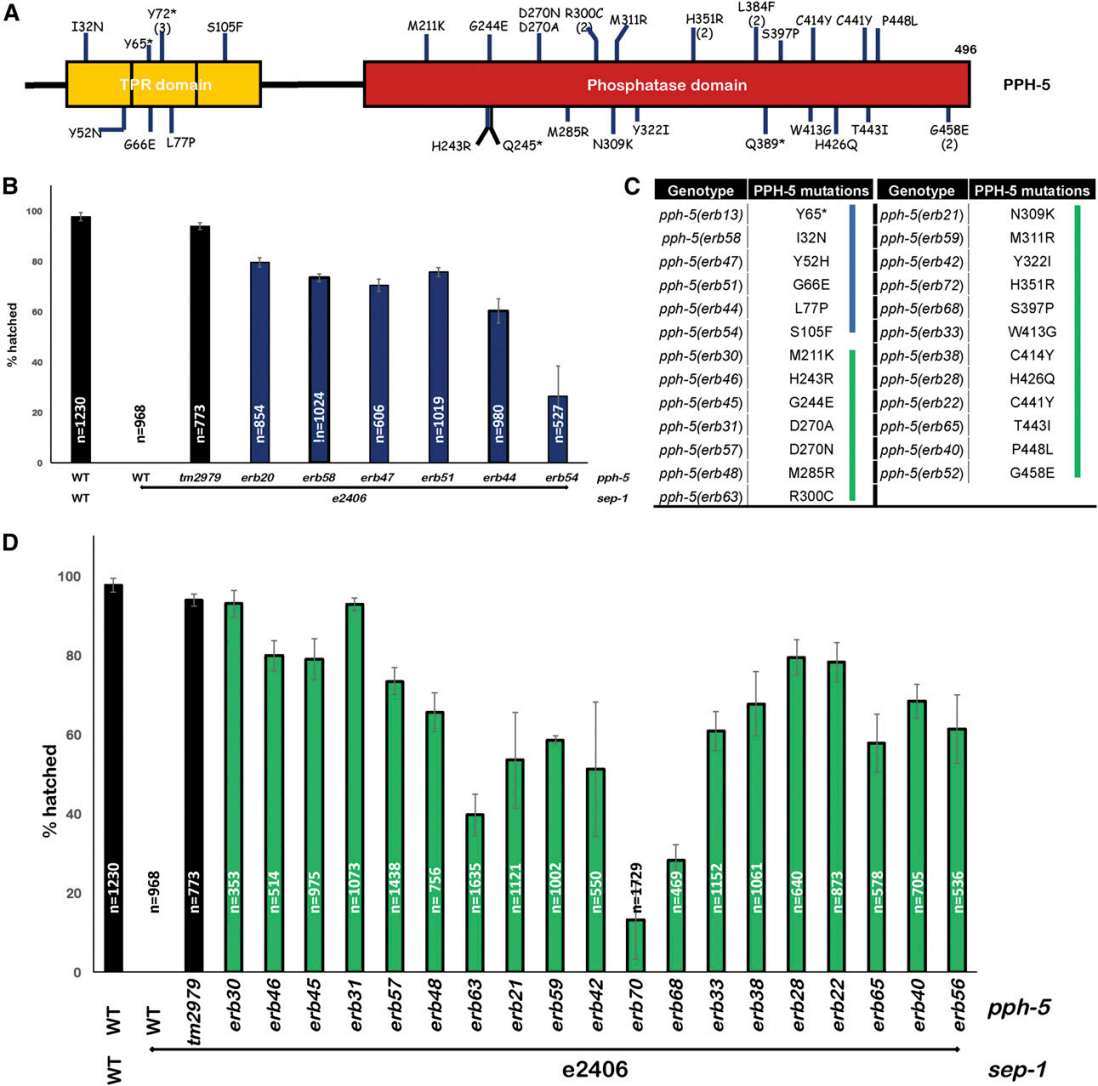
Mutations in *pph-5* are the most frequently identified *sep-1(e2406)* suppressors. (A) Protein diagram illustrating *pph-5* alleles suppressing *sep-1(e2406)*. These missense mutations span across PPH-5. Numbers in parentheses following a mutation indicate the number of times each suppressor was identified. * indicates nonsense mutations. This diagram does not depict splice site variants or frameshift mutations. (B) Embryonic lethality of *sep-1(e2406)* is rescued by missense mutations in the TPR domain of PPH-5, which might affect interactions with PPH-5 binding partners. (C) Table listing gene mutations and the resulting missense mutations in PPH-5. Blue and green bars indicate mutations in the TPR and the phosphatase domains of PPH-5, respectively. (D) Embryonic lethality of *sep-1(e2406)* is rescued by missense mutations in the phosphatase domain of PPH-5, which might affect catalytic activity (*n* = number of embryos). TPR, tetratricopeptide repeat; WT, wild-type.

The majority of *sep-1(e2406)* suppressors identified from our analysis are mutations in the protein phosphatase *pph-5*. The types of mutations identified include premature stop codons (12 alleles), splice site mutations (six alleles), as well as amino acid substitutions (29 alleles). Missense *pph-5*-suppressing mutations span the full length of the protein, altering both the TPR as well as the phosphatase domain. Excluding mutations that introduce a premature stop codon, our screen has identified 25 unique amino acid substitutions across the protein (Figure 3A). Missense suppressor mutations occur both within the TPR domain and the phosphatase domain of PPH-5, suggesting that both domains are required for PPH-5 regulation of separase. The capacity of these mutations to rescue *sep-1(e2406)* varies, as assayed by the proportion of embryos able to hatch at the restrictive temperature (Figure 3, B and D). Strong RNAi knockdown of *pph-5* (*pph-5 RNAi*) in these suppressed lines also results in improvement of suppression (Table 1). A nonsense allele, *erb13*, rescues hatching of *sep-1(e2406)* to 79%, which increases to 89% with *pph-5(RNAi)*, which is consistent with previous reports that *pph-5* null has little effect on viability. We also find that *pph-5(tm2979)* results in 94% hatching in *sep-1(e2406)*. These observations suggest that these suppressors are only partial reduction-of-function mutations and that suppression is more efficient with decreased PPH-5 activity. *pph-5* mutants are the most commonly isolated suppressors because mutations resulting in a reduction of PPH-5 function will suppress *sep-1(e2406)*.

**Table 1:** Reduction of *pph-5* by RNAi-mediated knockdown results in improved hatching

sep-1	*pph-5*	*pph-5 (RNAi)*		No RNAi
		Total Embryo	% Hatching	% Hatching
WT	WT	512	98.1	97.6
*e2406*	WT	578	72.8	0.0
	*erb13* (Y65*)	323	88.9	79.4
	*erb58* (I32N)	255	98.0	73.3
	*erb47* (Y52H)	235	73.5	70.4
	*erb51* (G66E)	589	77.1	75.7
	*erb44* (L77P)	226	95.1	60.2
	*erb54* (S105F)	298	33.9	26.3
	*erb30* (M211K)	113	94.7	92.9
	*erb46* (H243R)	215	85.1	79.8
	*erb45* (G244E)	604	78.3	78.9
	*erb31* (D270A)	320	94.7	92.8
	*erb57* (D270N)	434	97	73.4
	*erb48* (M285R)	325	91.1	65.6
	*erb63* (R300C)	322	75.5	39.6
	*erb21* (N309K)	351	95.4	53.4
	*erb59* (M311R)	378	65.5	58.6
	*erb42* (Y322I)	270	79.3	51.1
	*erb72* (H351R)	163	77.6	13.1
	*erb68* (S397P)	292	27.3	28.2
	*erb33* (W413G)	386	84.5	60.8
	*erb38* (C414Y)	255	76.5	67.7
	*erb28* (H426Q)	399	95.2	79.3
	*erb22* (C441Y)	374	79.1	78.1
	*erb65* (T443I)	399	84.4	57.7
	*erb40* (P448L)	109	74.3	68.4
	*erb52* (G458E)	282	78.0	61.3

RNAi knockdown of *pph-5* by feeding results in improved hatching efficiency in worms carrying *pph-5* mutations that suppress *sep-1(e2406)* lethality at the restrictive temperature of 20°. RNAi, RNA interference; WT, wild-type.

It has been shown that *pph-5* mutants do not suppress *sep-1(e2406)* by bypassing the separase requirement (Richie *et al.* 2011), as RNAi knockdown of *sep-1* still prevents chromosome segregation, causes cytokinesis failure, and results in lethality in *pph-5* mutants. *pph-5(av101)* also suppressed *sep-1(e2406)* at the restrictive temperature of 20° but not 24°, further indicating that *pph-5* mutants are probably not bypass suppressors because separase function is more compromised at higher temperature. *sep-1(e2406)* suppression by *pph-5* mutants (*av101* and *tm2979*) is semidominant and expected to be the case for the suppressors isolated here. The suppressors that we have identified are *pph-5* reduction-of-function mutants, which restore viability in a similar manner to previously identified *pph-5* mutants. Our data do not preclude the possibility that *pph-5* acts in a separase-independent pathway to restore viability to *sep-1(e2406)* animals. We favor our proposed model because mutations in *pph-5* have been demonstrated to restore mutant separase localization (Richie *et al.* 2011). One suppressor mutation in *pph-5* (L77P) was independently identified in both screens as a suppressor of conditional (*sep-1(e2406)*) and nonconditional (*sep-1(ax110)*) separase mutants. We also expect that the *sep-1(e2406)* suppressors that we have identified will suppress *sep-1(ax110)*, based on the observation that both *pph-5(tm2979)* and *pph-5(av101)* do not show *sep-1* allele-specific suppression at 20° (Richie *et al.* 2011). This extensive collection of *pph-5* mutants provides a valuable tool for structure–function, as well as genetic, analysis of this phosphatase.

### HSP-90 suppressor reveals a novel regulator of separase

**Figure 4:**
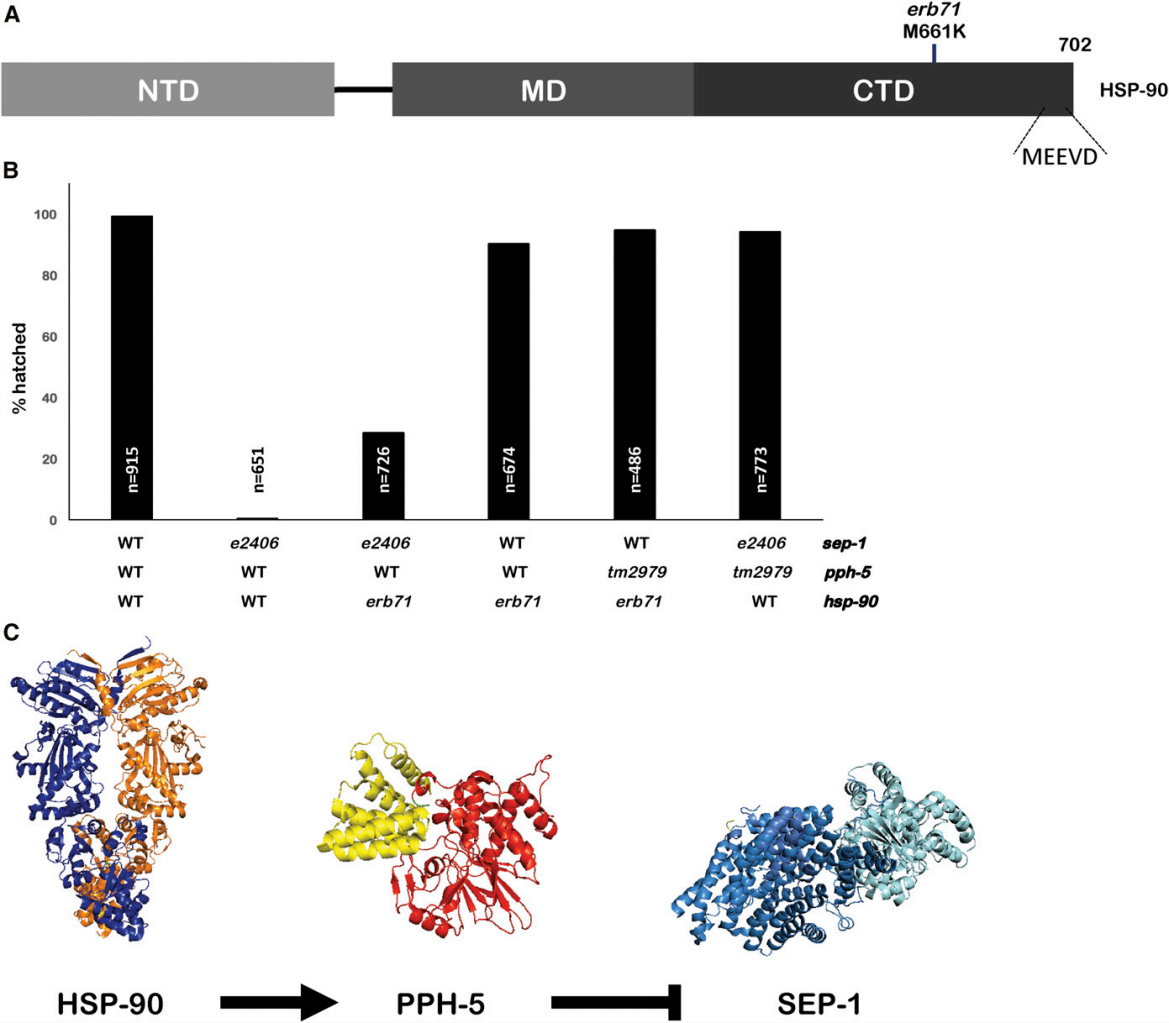
Mutation in the molecular chaperone *hsp-90* suppresses *sep-1(e2406)*. (A) Protein diagram of HSP-90. The *erb71* mutation results in a missense mutation at the C-terminal end of the protein chaperone HSP-90 (M661K), separated by 36 residues from the C-terminal MEEVD motif. HSP-90 protein domains are also illustrated (NTD, amino-terminal domain; MD, middle domain; CTD, carboxy-terminal domain; and MEEVD, Met-Glu-Glu-Val-Asp motif) (B) The *hsp-90(erb71)* mutant has a minimal effect on hatching when present in an otherwise wild-type (WT) background. Embryonic lethality is not reduced when *hsp-90(erb71)* is combined with a *pph-5* loss-of-function mutant (*n* = number of embryos). (C) Model for separase regulatory pathway: HSP-90 activates PPH-5 to negatively regulate separase function. Loss of this negative regulation suppresses *sep-1(e2406)*. Protein Data Bank structures modified from 5MZ6 (SEP-1), 4JA9 (PPH-5), and 5FWP (HSP-90).

The biochemical evidence connecting PPH-5 with HSP-90 (Haslbeck *et al.* 2015) prompted us to test if any of the suppressors were *hsp-90* mutations. We sequenced the *hsp-90* locus of the remaining suppressed lines that did not carry any suppressing intragenic or *pph-5* mutations. *Hsp-90* is essential in *C. elegans*, as null worms arrest growth at the L2–L3 stage (Birnby *et al.* 2000). We found that *erb71* has a single missense mutation, which changes methionine 661 into lysine (Figure 4A), that has an intermediate ability to restore hatching to 31% (Table 2). When isolated from *sep-1(e2406)*, the *hsp-90*(*erb71*) allele has a minimal effect on embryonic survival at 20° (84% hatching), which suggests that the essential functions of HSP-90 are minimally affected (Figure 4B). The rescue observed with *pph-5(RNAi)* is greater than the 31% survival observed in *hsp-90*(*erb71)*. This suggests that either the M661L mutation does not completely disrupt the PPH-5-activating functions of HSP-90 or that PPH-5 can still be active without HSP-90. Consistent with this, we observed improved survival (92.9% hatching) when *pph-5(RNAi)* was performed in a *sep-1(e2406)*; *hsp-90(erb71)* animal (Table 2). The identification of a HSP-90 allele that can suppress a temperature-sensitive separase mutation is consistent with the hypothesis that HSP-90 acts via its regulation of PPH-5. However, our data do not exclude the possibility that HSP-90 directly regulates separase independently of PPH-5.

**Table 2:** RNAi-mediated knockdown of *pph-5* in *hsp-90(erb71)* worms

Strain	Total Embryos	% Hatching
N2	512	98.4
N2, *pph-5(RNAi)*	352	97.8
*sep-1(e2406)*	223	0
*sep-1(e2406)*; *pph-5(RNAi)*	340	71.1
*sep-1(e2406)*; *hsp-90(erb71)*	181	30.9
*sep-1(e2406)*; *hsp-90(erb71)*; *pph-5(RNAi)*	157	92.9
*hsp-90(erb71)*	148	84.4
*hsp-90(erb71)*; *pph-5(RNAi)*	186	86.5

The genetic interaction between *pph-5* and *hsp-90* was investigated by using RNAi-mediated knockdown of *pph-5*. Reduction of PPH-5 in a worm carrying a *sep-1(e2406)*-rescuing *hsp-90* mutation results in reduced embryonic lethality at the restrictive temperature (20°). However, *pph-5(RNAi)* has little effect on the embryonic lethality of *hsp-90(erb71)*. RNAi, RNA interference.

Combining *hsp-90(erb71)* with *pph-5(RNAi)* has little effect on embryonic survival, compared to the effects of *erb71* alone (84% *vs.* 86% hatching) in an otherwise wild-type background. No significant changes in hatching were observed when *hsp-90(erb71)* was combined with *pph-5(tm2979)*. *pph-5(tm2979)* is an in-frame deletion that removes 55 amino acids from the PPH-5 TPR domain and potently suppresses *sep-1(e2406)* and *sep-1(ax110)* (Richie *et al.* 2011). These observations demonstrate that *pph-5* function is not critical, even in a mutant *hsp-90(erb71)* background, for the essential functions of HSP-90. Taken together, these observations support the hypothesis that *hsp-90(erb71)* does not result in a general loss in HSP-90 chaperone activity, and that we have isolated a rare loss-of-function allele that may specifically affect its interaction with PPH-5.

It is interesting to note that the mutation in HSP-90(erb71) (M661K) is found just N-terminal to the HSP-90 MEEVD motif, which is critical for HSP-90 to activate PPH-5 (Haslbeck *et al.* 2015). There is biochemical evidence that the PPH-5/HSP-90 interaction involves additional HSP-90 domains beyond the MEEVD motif. Activation of PPH-5 phosphatase by a peptide containing the MEEVD motif is less than that observed for full-length HSP-90 (Haslbeck *et al.* 2015). Cross-linking experiments also suggest additional contacts between HSP-90 and PPH-5. The corresponding residue mutated in *HSP-90(erb71)* in human HSP90 (M813) is part of the dimerization interface of two Hsp90 molecules near the site of a TPR-integrating MEEVD domain, as observed in a Cryo-EM structure (PDB 5FWP; Verba *et al.* 2016). This residue might alter the ability of the MEEVD peptide to bind to the TPR domain of PPH-5 by altering the C-terminal structure of HSP-90. Therefore, an analogous mutation in other organisms, such as humans, may be useful for studies of the HSP90-PP5 pathway. We propose a model for the regulation of separase, in which *pph-5* is a negative regulator of separase and PPH-5 activity is positively regulated by interactions with HSP-90 (Figure 4C). These new alleles of *hsp-90* and *pph-5* provide important tools for future dissection of this pathway.

### Novel sep-1(e2406) suppressors belong to multiple complementation groups

**Figure 5:**
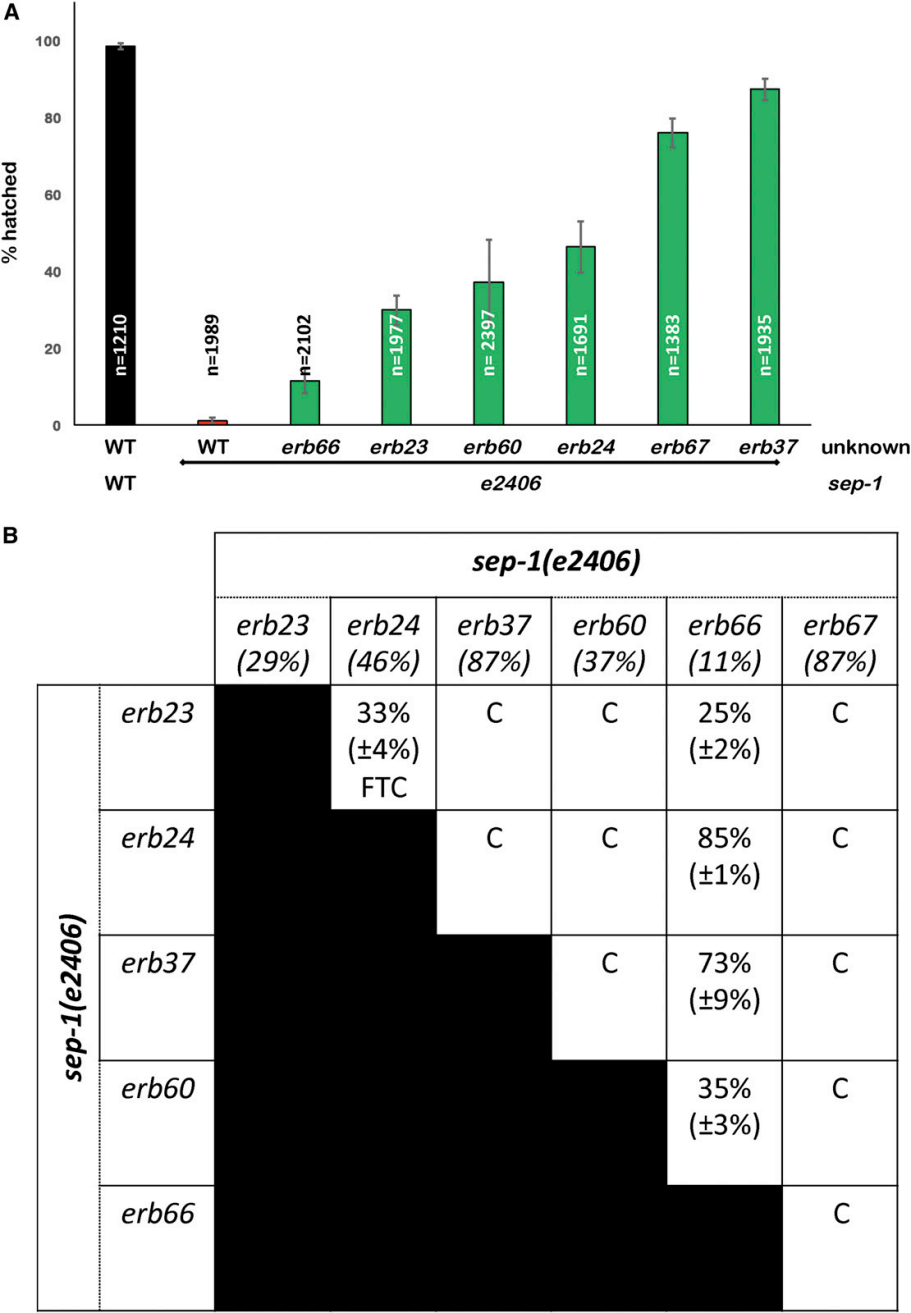
Novel suppressors of *sep-1(e2406)* belong to multiple complementation groups. (A) Strains carrying novel *sep-1(e2406)* suppressors result in varied rescue of embryonic lethality (*n* = number of embryos). (B) Complementation assay based on survival of F2 embryos of a cross between strains carrying novel *sep-1(e2406)* suppressors indicates that these suppressors belong to multiple complementation groups. Numbers below each parent strain or in a box representing a cross progeny indicate the percent of embryos that hatch at the restrictive temperature of 20°. The numbers in parentheses show the SD for three replicate hatching assays. C, complements; FTC, failure to complement.

Our suppressor screen identified six lines without suppressor mutations in the three genes that we sequenced (*sep-1*, *pph-5*, and *hsp-90*). These suppressed lines have varying degrees of hatching recovery at the restrictive temperature (Figure 5A). To determine the number of loci represented by this group of alleles, we performed pairwise complementation tests. The hatching efficiency of broods laid by F1 cross progeny between two homozygous suppressed lines was monitored at 20°. As presented in Figure 5B, these suppressors belong to four, possibly five, complementation groups. Two lines, *sep-1(e2406)*; *erb23* and *sep-1(e2406)*; *erb24*, do not complement and their cross progeny demonstrate an intermediate embryonic lethality as compared to the parents. Another mutant, *sep-1(e2406)*; *erb66*, appears to be dominant over other suppressors, except *erb67*, and cannot be assigned to a complementation group. Finally, *erb37*, *erb60*, and *erb67* do not result in suppression when crossed with other mutants and are likely mutations in three different genes. These observations provide an exciting opportunity to identify novel regulators of separase.

### Conclusions

By undertaking this extensive suppressor screen, we set out to identify separase regulators. Our results reveal that the phosphatase, *pph-5*, is a suppressor of *sep-1(e2406)* lethality. The results of our genetic screen highlight the importance of the *pph-5* regulatory pathway. The mechanism by which *pph-5* regulates separase during cytokinesis will be an important focus of future studies. Identification of the substrates of PPH-5 that become hyperphosphorylated in a *pph-5* mutant may elucidate this mechanism, as well as any additional roles that PPH-5 might play during mitosis. We have found that *hsp-90* also functions, likely via its regulation of *pph-5*, as a separase regulator. The sole *hsp-90* suppressor that we identified may be a rare hypomorphic mutant whose PPH-5 activating role is selectively reduced without compromising its other critical chaperone functions. Given the high degree of conservation of *pph-5* and *hsp-90*, we expect that our observations will be applicable to separase function in other systems as well. We were also able to identify novel intragenic suppressors, all of which are missense mutations in the N-terminal TPR-like domain of SEP-1, providing insight into this poorly characterized domain. TPR domains mediate protein–protein interactions and these residues may be involved in mediating interactions with separase binding partners that are required for its function. We have additionally isolated lines that carry mutations belonging to at least four complementation groups, giving us the opportunity to more extensively understand separase regulation. We will pursue a whole-genome sequencing approach to identify these mutations. This study demonstrates the power of genetics in understanding separase function and regulation.

## Supplementary Material

Supplemental material is available online at www.g3journal.org/lookup/suppl/doi:10.1534/g3.117.300298/-/DC1.
