# Supplementary material for: Genetic Identification of Separase Regulators in Caenorhabditis elegans
Source: G3 (Bethesda). 2017 Dec 14;8(2):695–705. doi: 10.1534/g3.117.300298 (PMC5919728; doi:10.1534/g3.117.300298)
Supplement: Supplementary file 1 [file 695FileS1.pdf]

Supplementary information

Figure S1: Illustration of suppressor mutations

Each residue mutated in a suppressed SEP-1 is illustrated in both its wild-type as well as mutant form. The residues within 6 angstroms of each residue are also depicted to provide a structural context for each mutation. A general trend observed is that the suppressor mutations introduce larger residues that have the potential to make new intramolecular contacts. Two alanine to valine mutations at positions 64 and 471 reduce the distance to other non-polar residues. A64V brings the distance to L376 from 5.6 to 4 Å and A417V lowers the distance to F476 and F454 from 4.6 and 5.6 Å to 3.3 and 4.4 Å, respectively. Two mutations that replace polar residues with isoleucine have the potential to create new hydrophobic interactions. T357I brings the residue to within 4.2 Å and N517I brings the residue within 4.8 Å of I552 and 4.7 Å of L556. A mutation that preserves residue charge (D541E) reduces the distance from the basic residue (K482) from 4.3 to 4.2 Å. L556 mutations to histidine creates the potential for a new interaction with E160 while L556F brings R551 within 2.5 Å, potentially allowing a cation-π interaction. Images and measurements were generated using PyMOL Molecular Graphics System from Schrödinger, LLC.

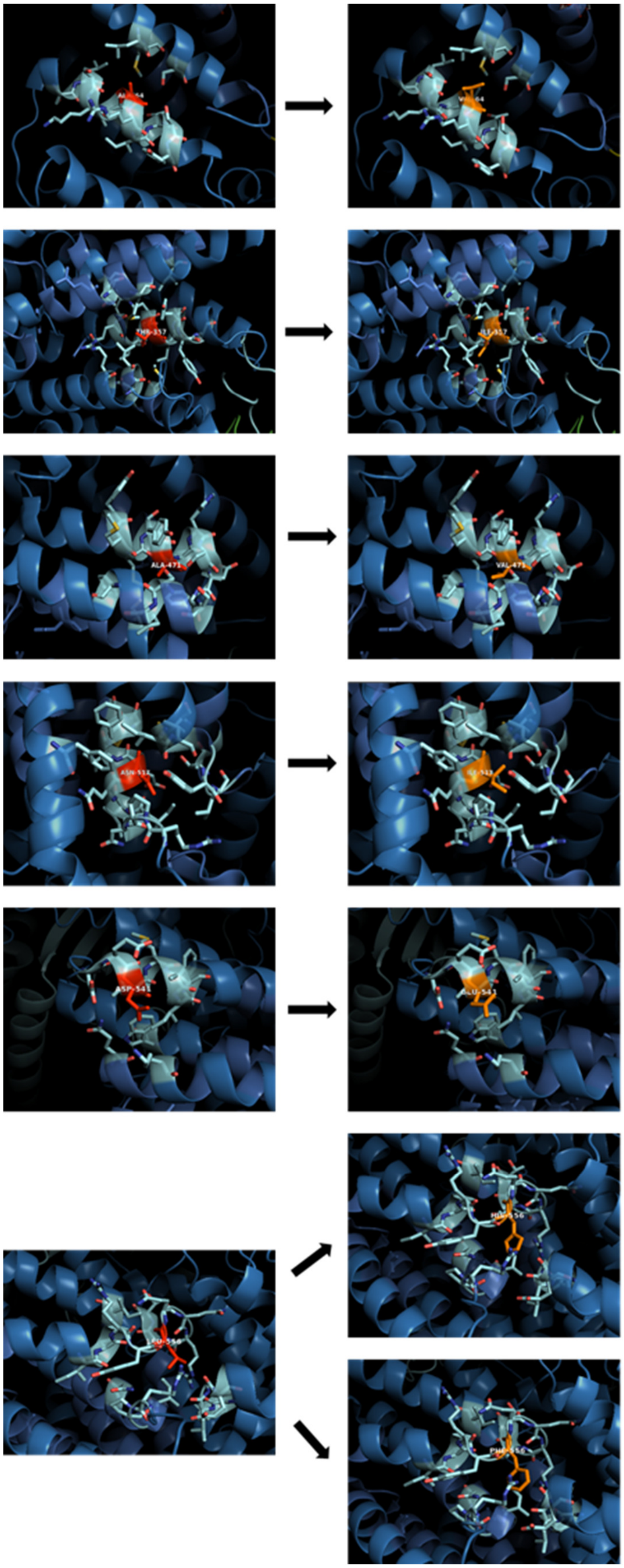

A64V

T357I

A471V

N517I

D541E

L556F

L556H

17

Table S1: List of Strains

Strain	Mutation	Genotype	Isolation Frequency
WH408	SEP-1 (C450Y)	*sep-1(e2406)* I/*hT2g [bli-4(e937) let-?(q782)* qIs48*]* (I;III)	N/A
JAB54	SEP-1 (C450Y A64V)	*sep-1(e2406erb14)* I	2
JAB53	SEP-1 (C450Y T357I)	*sep-1 (e2406erb16)* I	1
JAB76	SEP-1 (C450Y V392I)	*sep-1(e2406erb27)* I	1
JAB126	SEP-1 (C450Y A471V)	*sep-1(e2406erb29)* I	1
JAB50	SEP-1 (C450Y N517I)	*sep-1 (e2406erb17)* I	1
JAB58	SEP-1 (C450Y D541E)	*sep-1(e2406erb15)* I	1
JAB51	SEP-1 (C450Y L556H)	*sep-1 (2406erb10)* I	2
JAB45	SEP-1 (C450Y L556F)	*sep-1(e2406erb5)* I	4
JAB46	SEP-1 (C450Y L556F)	*sep-1(e2406erb6)* I	4
JAB47	SEP-1 (Y450C)	*sep-1(erb7)* I	1
JAB48	SEP-1 (C450Y L556F)	*sep-1(e2406erb8)* I	4
JAB49	SEP-1 (C450Y L556F)	*sep-1(e2406erb9)* I	4
JAB61	SEP-1 (C450Y L556H)	*sep-1(e2406erb18)* I	2
JAB64	SEP-1 (C450Y A64V)	*sep-1(e2406erb19)* I	2
JAB106	SEP-1 (C450Y); PPH-5 (I32N)	*sep-1(e2406)* I; *pph-5(erb58)* V	1
JAB97	SEP-1 (C450Y); PPH-5 (Y52H)	*sep-1(e2406)* I; *pph-5(erb47)* V	1
JAB99	SEP-1 (C450Y); PPH-5 (G66E)	*sep-1(e2406)* I; *pph-5(erb51)* V	1
JAB65	SEP-1 (C450Y); PPH-5 (Y72*)	*sep-1(e2406)* I; *pph-5(erb20)* V	2
JAB94	SEP-1 (C450Y); PPH-5 (L77P)	*sep-1(e2406)* I; *pph-5(erb44)* V	1
JAB103	SEP-1 (C450Y); PPH-5 (S105F)	*sep-1(e2406)* I; *pph-5(erb54)* V	1
JAB79	SEP-1 (C450Y); PPH-5 (M211K)	*sep-1(e2406)* I; *pph-5(erb30)* V	1
JAB96	SEP-1 (C450Y); PPH-5 (H243R)	*sep-1(e2406)* I; *pph-5(erb46)* V	1
JAB95	SEP-1 (C450Y); PPH-5 (G244E)	*sep-1(e2406)* I; *pph-5(erb45)* V	1
JAB80	SEP-1 (C450Y); PPH-5 (D270A)	*sep-1(e2406)* I; *pph-5(erb31)* V	1
JAB67	SEP-1 (C450Y); PPH-5 (D270N)	*sep-1(e2406)* I; *pph-5(erb57)* V	1
JAB98	SEP-1 (C450Y); PPH-5 (M285R)	*sep-1(e2406)* I; *pph-5(erb48)* V	1
JAB111	SEP-1 (C450Y); PPH-5 (R300C)	*sep-1(e2406)* I; *pph-5(erb63)* V	2
JAB66	SEP-1 (C450Y); PPH-5 (N309K)	*sep-1(e2406)* I; *pph-5(erb21)* V	1
JAB107	SEP-1 (C450Y); PPH-5 (M311R)	*sep-1(e2406)* I; *pph-5(erb59)* V	1
JAB91	SEP-1 (C450Y); PPH-5 (Y322I)	*sep-1(e2406)* I; *pph-5(erb42)* V	1
JAB120	SEP-1 (C450Y); PPH-5 (S397P)	*sep-1(e2406)* I; *pph-5(erb68)* V	1
JAB72	SEP-1 (C450Y); PPH-5 (W413G)	*sep-1(e2406)* I; *pph-5(erb33)* V	1
JAB88	SEP-1 (C450Y); PPH-5 (C414Y)	*sep-1(e2406)* I; *pph-5(erb38)* V	1
JAB77	SEP-1 (C450Y); PPH-5 (H426Q)	*sep-1(e2406)* I; *pph-5(erb28)* V	1
JAB70	SEP-1 (C450Y); PPH-5 (C441Y)	*sep-1(e2406)* I; *pph-5(erb22)* V	1
JAB117	SEP-1 (C450Y); PPH-5 (T443I)	*sep-1(e2406)* I; *pph-5(erb65)* V	1

JAB89	SEP-1 (C450Y); PPH-5 (P448L)	*sep-1(e2406)* I; *pph-5(erb40)* V	1
JAB100	SEP-1 (C450Y); PPH-5 (G458E)	*sep-1(e2406)* I; *pph-5(erb52)* V	2
JAB121	SEP-1 (C450Y); PPH-5 (A471*)	*sep-1(e2406)* I; *pph-5(erb69)* V	1
JAB122	SEP-1 (C450Y); PPH-5 (H351R)	*sep-1(e2406)* I; *pph-5(erb70)* V	2
JAB124	SEP-1 (C450Y); PPH-5 (H351R)	*sep-1(e2406)* I; *pph-5(erb72)* V	2
JAB55	Splice site acceptor (*pph-5* intron 6)	*sep-1(e2406)* I; *pph-5(erb11)* V	1
JAB56	Splice site donor (*pph-5* intron 3)	*sep-1(e2406)* I; *pph-5(erb12)* V	1
JAB57	SEP-1 (C450Y); PPH-5 (Y65* Y72*)	*sep-1(e2406)* I; *pph-5(erb13)* V	1
JAB73	250 bp deletion (*pph-5* exon 1-2)	*sep-1(e2406)* I; *pph-5(erb25)* V	1
JAB75	Splice site donor (*pph-5* intron 1)	*sep-1(e2406)* I; *pph-5(erb26)* V	1
JAB85	32bp deletion (*pph-5* exon 1)	*sep-1(e2406)* I; *pph-5(erb32)* V	1
JAB62	SEP-1 (C450Y A471V); PPH-5 (L384F)	*sep-1(e2406 erb29)* I; *pph-5(erb34)* V	1
JAB86	Repetitive region of *pph-5*	*sep-1(e2406)* I; *pph-5(erb35)* V	1
JAB63	SEP-1 (C450Y); PPH-5 (Y72*)	*sep-1(e2406)* I; *pph-5(erb36)* V	2
JAB69	Splice site acceptor (*pph-5* intron 7)	*sep-1(e2406)* I; *pph-5(erb39)* V	1
JAB90	SEP-1 (C450Y); PPH-5 (Q245*)	*sep-1(e2406)* I; *pph-5(erb41)* V	1
JAB93	SEP-1 (C450Y); PPH-5 (Q389*)	*sep-1(e2406)* I; *pph-5(erb43)* V	1
JAB125	Deletion of *pph-5* exon 4 & 5	*sep-1(e2406)* I; *pph-5(erb49)* V	1
JAB127	Deletion of *pph-5* exon 5	*sep-1(e2406)* I; *pph-5(erb50)* V	1
JAB102	SEP-1 (C450Y); PPH-5 (R300C)	*sep-1(e2406)* I; *pph-5(erb53)* V	1
JAB104	Splice site acceptor (*pph-5* intron 1)	*sep-1(e2406)* I; *pph-5(erb55)* V	1
JAB105	SEP-1 (C450Y); PPH-5 (G458E)	*sep-1(e2406)* I; *pph-5(erb56)* V	2
JAB109	98bp deletion (*pph-5* exon 3)	*sep-1(e2406)* I; *pph-5(erb61)* V	1
JAB110	Splice site acceptor (*pph-5* intron 1)	*sep-1(e2406)* I; *pph-5(erb62)* V	1
JAB112	SEP-1 (C450Y); PPH-5 (L384F)	*sep-1(e2406)* I; *pph-5(erb64)* V	1
JAB123	SEP-1 (C450Y); HSP-90 (M661K)	*sep-1(e2406)* I; *hsp-90(erb71)* V	1
JAB136	HSP-90 (M661K)	*hsp-90(erb71)* V	N/A
JAB78	SEP-1 (C450Y); unk	*sep-1(e2406)* I; *erb23*	1
JAB59	SEP-1 (C450Y); unk	*sep-1(e2406)* I; *erb24*	1
JAB87	SEP-1 (C450Y); unk	*sep-1(e2406)* I; *erb37*	1
JAB108	SEP-1 (C450Y); unk	*sep-1(e2406)* I; *erb60*	1
JAB118	SEP-1 (C450Y); unk	*sep-1(e2406)* I; *erb66*	1
JAB119	SEP-1 (C450Y); unk	*sep-1(e2406)* I; *erb67*	1
WH410	SEP-1 (H738P)	*sep-1(ax110)* I/hT2g *[bli-4(e937) let-?(q782)* qIs48*]* (I;III)	N/A
JAB42	SEP-1 (H738P); PPH-5(S229L)	*sep-1(ax110)* I; *pph-5(erb1)* V	1
JAB43	SEP-1 (H738P); PPH-5(M380T)	*sep-1(ax110)* I; *pph-5(erb2)* V	1
JAB44	SEP-1 (H738P); PPH-5(L77P)	*sep-1(ax110)* I; *pph-5(erb3)* V	2

JAB92	SEP-1 (H738P); PPH-5(L77P)	*sep-1(ax110)* I; *pph-5(erb4)* V	2

Table S2: List of primers used for single worm PCR and sequencing of *sep-1*

Primer	Target	Sequence*	Direction
oASP-UTK3	*sep-1(ax110)*	GTCGTCCGAAAGTTGAGAAACTGG	F
oASP-UTK4	*sep-1(ax110)*	TGCATCAGCGGCAGGAACGATT	R
oASP-UTK7	*sep-1(e2406)*	ctgagacatcacacgaaagc	F
oASP-UTK8	*sep-1(e2406)*	GAGTTGTCGTCCAAGTTGTG	R
oASP-UTK9	*sep-1* FL	ATGAAGATCACAAACAAGTCAG	F
oASP-UTK10	*sep-1* FL	TTACAAATTTCGGGATCTTGATGG	R
oASP-UTK11	*sep-1* FL-seq	AATCGTTCCTGCCGCTGATGCA	F
oASP-UTK12	*sep-1* FL-seq	cagCTTTATTGGACATGGCTCTGGA	F
oASP-UTK13	*sep-1* exon 1	gaagataacgtgcgttgaaacc	F
oASP-UTK14	*sep-1* exon 3	ccgtctctgtagggtcaaattc	R
oASP-UTK28	*sep-1(ax110)*	aaaatcaaattctaccgagcg	F
oASP-UTK29	*sep-1(ax110)*	gcatcaaataggttgcgaaatac	R
oASP-UTK30	*sep-1(ax110)*	GTCGTCCGAAAGTTGAGAAACT	F
oASP-UTK34	*sep-1(e2406)*	gtagatttacggcgctttgc	F
oASP-UTK46	*sep-1* exon 4	ctgagacatcacacgaaagcc	F
oASP-UTK47	*sep-1* exon 8	AGAGCCATGTCCAATAAAGctg	R
oASP-UTK48	*sep-1* exon 7	GAGAAGTTCAAATCGTTCCTGCC	F
oASP-UTK54	*sep-1* seq primer	TCCAGAGCCATGTCCAATAAAGctg	R

* nucleotides found in exons are in uppercase while intronic nucleotides are in lowercase

Table S3: List of primers used for single worm PCR and sequencing of *pph-5*

Primer	Target	Sequence*	Direction
oASP-UTK15	*pph-5* exon1-2	ggttaaattttagggaaaccgc	F
oASP-UTK16	*pph-5* exon1-2	cgaaattttctcttaaaccggg	R
oASP-UTK17	*pph-5* exon 3	gctgctaattcaattttgggc	F
oASP-UTK18	*pph-5* exon 3	tgccggttttacatcaattttc	R
oASP-UTK19	*pph-5* exon 4	gaaaacgtgcaaatttccaatc	F
oASP-UTK20	*pph-5* exon 4	cctcaattttcagctgttttgg	R
oASP-UTK21	*pph-5* exon 5	gaaaatggccaaattttcac	F
oASP-UTK22	*pph-5* exon 5	aggatttcagcgatttctgg	R
oASP-UTK24	*pph-5* exon 6	cagattattgattttcccgacag	R
oASP-UTK26	*pph-5* exon 8	ctgaaattgcttgtaaagcctc	F
oASP-UTK27	*pph-5* exon 8	tggaagagattttcgtttcgtg	R
oASP-UTK35	*pph-5* exon 7	tgatttcagctcaaaattaacg	F
oASP-UTK37	*pph-5* exon 7	cagtgcgatttcttacCGTC	R
oASP-UTK38	*pph-5* exon 6	cagGTGTGCCATGGTGGATT	F
oASP-UTK39	*pph-5* exon 6	cagccattttcctacaaacctacC	R

oASP-UTK40	*pph-5* exon 8	caattttcagCCCCGATTCACG	F
oASP-UTK41	*pph-5* exon 8	taggcctaactcggcCTAATT	R
oASP-UTK50	*pph-5* exon 5	Aggatttcagcgatttctgggc	R
oASP-UTK51	*pph-5* exon 6	ggtatttttccagGTGTGCCATGG	F
oASP-UTK52	*pph-5* exon 7	gcagtgcgatttcttacCGTC	R
oASP-UTK53	*pph-5* exon 5	ccgaaaatggccaaattttcac	F

* nucleotides found in exons are in uppercase while intronic nucleotides are in lowercase

Table S4: List of primers used for single worm PCR and sequencing of *hsp-90*

Primer	Target	Sequence*	Direction
oASP-UTK60	*hsp-90*	ATGTCCGAGAACGCCGAAAC	F
oASP-UTK61	*hsp-90*	aggatgtTTAGTCGACCTCCTCC	R
oASP-UTK62	*hsp-90* seq	GTATTCTGGCATGAGCTCTTCG	R
oASP-UTK63	*hsp-90* seq	CACAAAGAGCTCCATTTGATCTC	F

* nucleotides found in exons are in uppercase while intronic nucleotides are in lowercase

Table S5: List of all *sep-1(e2406)* suppressing *pph-5* lesions

**Pph-5 allele**	**Nucleotide change**	**Effect**
pph-5(erb11)	G>A	Splice site acceptor intron 6
pph-5(erb12)	30 bp deletion spanning exon 3 and intron 3	splice site donor intron 3
pph-5(erb13)	G>A, T>A	Y65*, Y72*
pph-5(erb20)	T>G	Y72*
pph-5(erb21)	G>A	N309K
Pph-5(erb22)	G>A	C441Y
pph-5(erb25)	250 bp deletion in exon 1-2	Frameshift, early stop
pph-5(erb26)	T>A	Splice site donor exon 1
pph-5(erb28)	T>A	H426Q
pph-5(erb30)	T>A	M211K
pph-5(erb31)	A>C	D270A
pph-5(erb32)	32 bp deletion in exon1	Frameshift, early stop
pph-5(erb33)	T>G	W413G
pph-5(erb34)	C>T	L384F
pph-5(erb36)	T>G	Y72*
pph-5(erb38)	G>A	C414Y
pph-5(erb39)	G>A	Splice site acceptor, exon 7
pph-5(erb40)	C>T	P448L
pph-5(erb41)	C>T	Q245*
pph-5(erb42)	1 bp deletion in exon 5	Y322I, Frameshift early stop

pph-5(erb43)	C>T	Q389*
pph-5(erb44)	C>T	L77P
pph-5(erb45)	G>A	G244E
pph-5(erb46)	A>G	H243R
pph-5(erb47)	T>A	Y52H
pph-5(erb48)	T>G	M285R
pph-5(erb49)	Deletion	Loss of exon 4 and 5
pph-5(erb50)	Deletion	Loss of exon 5
pph-5(erb51)	G>A	G66E
pph-5(erb52)	C>T	G458E
pph-5(erb53)	C>T	R300C
pph-5(erb54)	C>T	S105F
pph-5(erb55)	G>A	Splice site acceptor exon 2
pph-5(erb56)	G>A	G458E
pph-5(erb57)	G>A	D270N
pph-5(erb58)	T>A	I32N
pph-5(erb59)	T>G	M311R
pph-5(erb61)	98 bp deletion in exon 3	Frameshift early stop
pph-5(erb62)	G>A	Splice site acceptor exon 2
pph-5(erb63)	C>T	R300C
pph-5(erb64)	C>T	L384F
pph-5(erb65)	C>T	T443I
pph-5(erb68)	T>C	S397P
